# Congenital Diaphragmatic Hernia with Intrathoracic Renal Ectopia: Thoracoscopic Approach for a Complete Anatomical Repair

**DOI:** 10.1055/s-0039-3402741

**Published:** 2020-10-21

**Authors:** Colin Mizzi, David Farrugia, Muhammad S. Choudhry

**Affiliations:** 1 Department of Paediatric Surgery, Mater Dei Hospital, Msida, Malta; 2 Department of Urology, Mater Dei Hospital, Msida, Malta; 3 Department of Paediatric Surgery, Chelsea and Westminster Hospital NHS Foundation Trust, London, United Kingdom of Great Britain and Northern Ireland

**Keywords:** diaphragmatic hernia, renal ectopia, intrathoracic kidney

## Abstract

Congenital diaphragmatic herniae (CDH) with associated intrathoracic ectopic kidneys are rare congenital anomalies, with a reported incidence of only 0.25%. The authors report a case of a 24-day-old baby girl who was diagnosed with a left-sided CDH on a chest X-ray taken for pneumonia. Computed tomography scan showed CDH hernia, containing small and large bowel and whole left kidney with adrenal gland. Thoracoscopic reduction in the bowel, kidney, and adrenal gland into the abdomen and primary closure of the defect was achieved with no complications. During investigation of the child, it was discovered that her maternal aunt had also had a left-sided congenital diaphragmatic hernia containing the kidney, which was treated via open surgery after birth; she subsequently developed renal cell carcinoma and required radical nephrectomy of that kidney during her third decade.

## Introduction

Congenital diaphragmatic hernias (CDH)occur in 1 of 2000 to 3000 live births, the commonest being Bochdalek (posterolateral) on the left side, 1 with less than 2% being familial cases. Isolated renal ectopia has an incidence of up to 1 in 500 cases on autopsy studies; intrathoracic renal ectopia is, however, the rarest form, having a prevalence of less than 0.01%. 2 The incidence of Bochdalek hernia with associated intrathoracic renal ectopia was reported at 0.25% 3 it is commoner in males and usually asymptomatic. 4 A single report of renal cell carcinoma occurring in kidney that had previously been in the thorax was found in the literature. 5

## Case Presentation

We report a 24-day-old female baby who presented with dyspnea and fever. Examination showed decreased air entry on the left side and a subsequent chest radiograph showed what looked like a consolidation of that lung and air-filled bowel loops inside the chest and a further ultrasound examination also identified intrathoracic ipsilateral kidney. A computed tomography (CT) of her thorax was performed, which confirmed left-sided diaphragmatic defect with large and small bowel and a normal looking, completely intrathoracic, ipsilateral left kidney and adrenal gland. The baby was delivered by spontaneous vaginal delivery with no antenatal diagnosis; she had normal Apgar score and did not require any resuscitation.

**Fig. 1:**
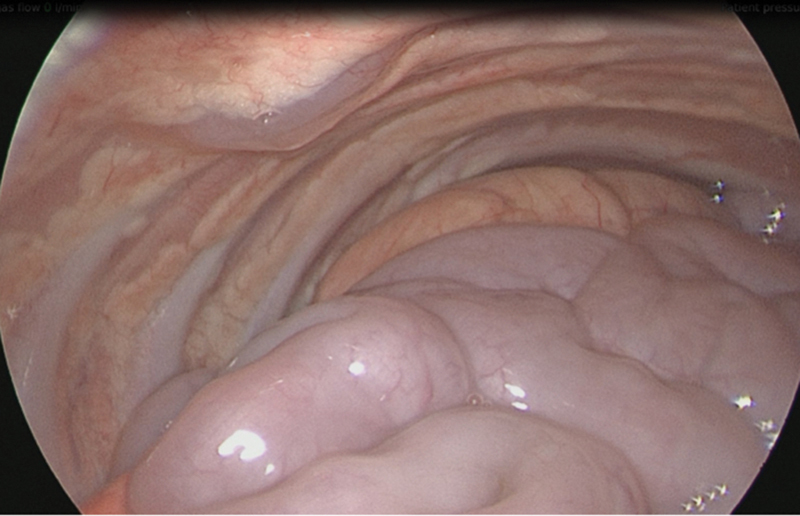
Intrathoracic kidney along with mutiple loops of small bowel.

**Fig. 2:**
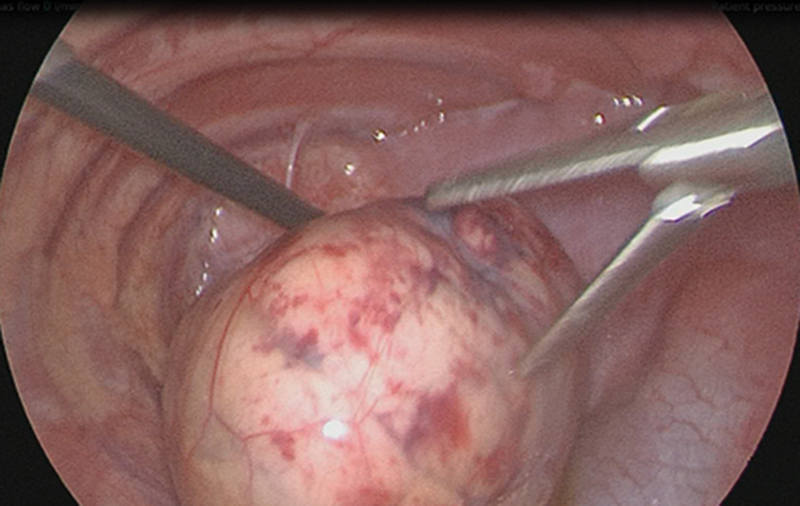
Reduction of intrathoracic kidney.

The procedure was performed in left lateral decubitus position. A 5 mm camera port was inserted by open technique in anterior axillary and two 3 mm working ports were inserted under vision on either side of camera port. The examination confirmed the CT findings ( Fig. 1 ). The herniated large and small bowel was pushed and reduced into the abdominal cavity through the diaphragmatic defect which then revealed a completely intrathoracic kidney encased in Gerota's fascia ( Fig. 2 ).

**Fig. 3:**
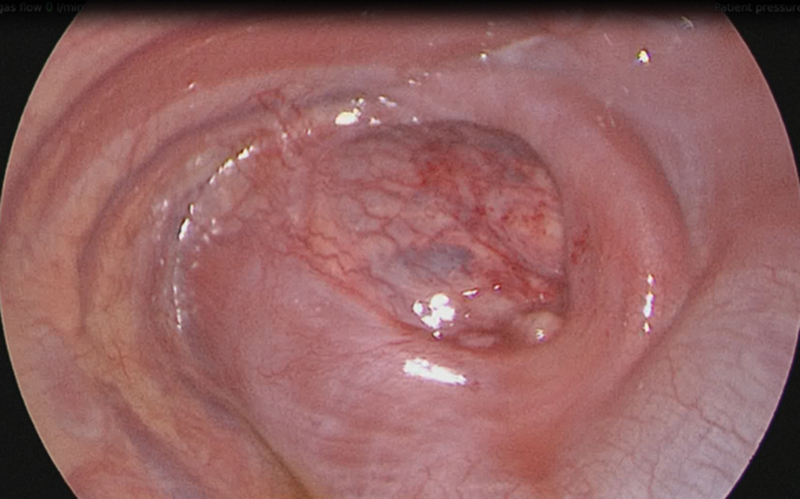
Posterolateral diaphragmatic defect.

**Fig. 4:**
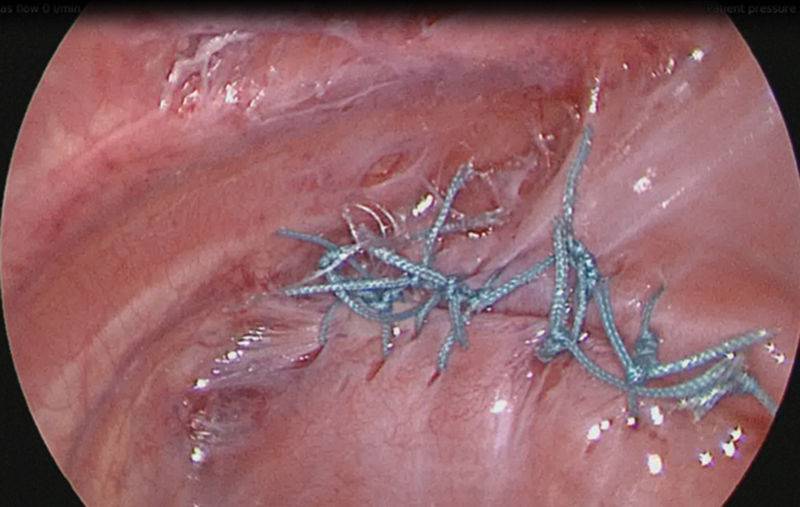
Primary closure of diaphragmatic defect.

The kidney was then reduced into the abdominal cavity retroperitoneally by pushing it down through the diaphragmatic defect ( Fig. 3 ). A primary repair using interrupted nonabsorbable sutures was achieved without tension ( Fig. 4 ). Initially the gas pressure during the reduction in hernia contents was kept at 6 mm Hg but was then reduced to 4 mm Hg during closure of the defect. A chest drain was left in situ through the 5 mm port site at the end of procedure as there was violation of the pleura.

The baby had uneventful recovery and was extubated within 24 hours and chest drain was removed after 48 hours. Renal ultrasound scan showed a correctly positioned kidney with good perfusion and no signs of obstruction. She was discharged on day 5 postoperatively and is being followed up as an outpatient; her most recent renal ultrasound scan done 3 years postoperatively showed well and equally perfused kidneys with comparable renal volumes bilaterally without any evidence of hydronephrosis or hydroureter, or recurrence of the diaphragmatic hernia.

During admission, the patient's mother gave a similar family history—her sister had had a similar condition at birth more than three decades ago. She had open repair of her diaphragmatic hernia at which an intrathoracic kidney was also found and reduced into the abdominal cavity. In the previous months, she had been diagnosed with renal cell carcinoma in that kidney and she had just undergone a left radical nephrectomy for her condition. The child's parent asked whether she might have an increased risk of having similarly effected children and whether her daughter now had a higher risk of developing renal cell carcinoma in this kidney.

## Discussion

Bochdalek hernias are postulated to occur due to delayed fusion of the pleuroperitoneal membranes with the septum transversum at 8 weeks' gestation; they are commoner on the left and in males. 6 Familial cases are extremely rare, accounting for less than 2% of all cases of CDH. 1 With a reported mortality of up to 30%, mainly due to pulmonary hypoplasia, large defects present as emergencies in the neonatal period, requiring urgent reduction in the abdominal organs into the abdominal cavity and closure of the defect. This can be done via open thoracotomy or minimally invasive thoracoscopic repair.

Intrathoracic renal ectopia is a rare entity, commoner in males, and on the left side—possibly due to earlier closure of Bochdalek canal and the position of the liver on the right side. Intrathoracic kidneys are classified into four groups: (1) intact diaphragm, (2) eventration of the diaphragm, (3) diaphragmatic hernia—congenital or acquired, and (4) traumatic diaphragmatic rupture. 7 It has been postulated that embryologically, intrathoracic renal ectopia may be related either to accelerated ascent due to delayed involution of the mesonephros or due to delayed closure of the pleuroperitoneal membranes. 8 Intrathoracic kidneys are generally diagnosed incidentally, having normal function; common anatomical anomalies associated with them are an elongated ureter, high origin of the vessels, abnormal rotation, and medial deviation of the inferior pole 3 ; though there might be an increased tendency to reflux and stone formation, intrathoracic kidneys generally follow a benign course if left untouched. 4 An intrathoracic kidney with concomitant CDH may be caused by direct herniation through the diaphragmatic defect into the intrathoracic space or by primary location in the posterior mediastinum due to a true embryologic ectopia.

Several studies have assessed the effectiveness of thoracoscopic repair of CDH compared with open repair 9 10 11 —in all studies reviewed it was found that thoracoscopic repair is at least as effective as open repair, with decreased postoperative morbidity and hospital length of stay.

In our case, the patient had a second-degree relative that had a similar history with a CDH and intrathoracic renal ectopy diagnosed and repaired when young; however, this lady subsequently developed renal cell carcinoma in the effected kidney requiring surgery at a young age in the third decade of life. Unfortunately, no genetic studies were done on the second-degree relative, so unfortunately one could not conclude that this is due to chance or due to an underlying mechanism. Literature search for any link between renal ectopy and increased risk of malignancy did not yield any results. There have been only nine reports of malignancy arising in an ectopic kidney since computed tomography was introduced 5 9 10 11 12 13 14 15 16 Of these, only one case was reported where papillary renal cell carcinoma occurred in an intrathoracic kidney and the patient in question had end-stage kidney disease and had undergone a renal transplant 6 years before, putting her at an increased risk of malignancy in general.

Though several genetic defects have been linked to renal developmental anomalies and syndromes associated with an increased risk of developing renal malignancy, no specific genetic mutation linking intrathoracic renal ectopy, CDH, and increased risk of renal cell carcinoma has been documented.

## Conclusion

We report a successful repair of CDH in a baby with ipsilateral ectopic kidney using a minimally invasive thoracoscopic approach with a similar family history that in due course developed renal carcinoma. This allows satisfactory reduction in the kidney in the retroperitoneal space and a complete anatomical repair.

## Notes

New Insights & the Importance for the Pediatric Surgeon The thoracoscopic approach is an alternative approach for the reduction in intrathoracic kidney.
